# Shengqing Jiangzhuo Capsule Alleviates Intestinal Inflammation in Chronic Kidney Disease by Downregulating CHAC1 to Inactivate the HIF-1 Pathway

**DOI:** 10.1155/mi/2173234

**Published:** 2025-11-06

**Authors:** Zhibin Li, Jian Wang, Yanna Lin, Shu Zhang, Liangyou Zhang, Xingbo Wu, Chao Wang

**Affiliations:** ^1^Department of Blood Purification, The First Affiliated Hospital of Guangzhou University of Chinese Medicine, Guangdong Clinical Research Academy of Chinese Medicine, No. 16, Airport Road, Guangzhou City 510400, Guangdong Province, China; ^2^Department of Comprehensive (VIP) Inpatient Ward, Sichuan Clinical Research Center for Cancer, Sichuan Cancer Hospital and Institute, Sichuan Cancer Center, University of Electronic Science and Technology of China, No. 55, Section 4, Renmin South Road, Wuhou District, Chengdu City 610000, Sichuan Province, China; ^3^Department of Oncology, Luzhou People's Hospital, Luzhou City 646000, Sichuan Province, China; ^4^Department of Continuing Education, The First Affiliated Hospital of Guangzhou University of Chinese Medicine, Guangdong Clinical Research Academy of Chinese Medicine, No. 16, Airport Road, Guangzhou City 510400, Guangdong Province, China; ^5^Department of Nephrology, The First Affiliated Hospital of Guangzhou University of Chinese Medicine, Guangdong Clinical Research Academy of Chinese Medicine, No. 16, Airport Road, Guangzhou City 510400, Guangdong Province, China

**Keywords:** CHAC1, chronic kidney disease, intestinal inflammation, Shengqing Jiangzhuo capsule, the HIF-1 signaling pathway

## Abstract

**Background:**

Chronic kidney disease (CKD) imposes significant global health burdens. Shengqing Jiangzhuo (SQJZ) capsule possesses potential to alleviate CKD via gut-kidney axis, with the specific role and mechanisms involving CHAC glutathione-specific γ-glutamylcyclotransferase 1 (CHAC1) and hypoxia-inducible factor 1 (HIF-1) signaling remaining unclear.

**Methods:**

Adenine-induced CKD rats were treated with SQJZ capsule for 4 weeks. The levels of blood urea nitrogen (BUN), serum creatinine (SCR), urine albumin/creatinine ratio (ACR), and inflammatory markers in colon tissues, including proinflammatory cytokines and oxidative markers, were assessed via enzyme-linked immunosorbent assay (ELISA). The renal pathology was estimated by histopathology. Transcriptomic sequencing combined with bioinformatics analysis identified the downstream pathway regulated by SQJZ in colon tissues. In vitro, after treatment with CHAC1 knockdown or HIF-1α activation, lipopolysaccharide (LPS)-treated NCM460 cells were analyzed for apoptosis, detected by flow cytometry, and inflammatory marker levels, determined by ELISA.

**Results:**

SQJZ significantly reduced serum BUN, SCR, and urinary ACR in CKD rats, ameliorating histopathological damage. In colon tissues, SQJZ suppressed proinflammatory cytokines, including interleukin-1β (IL-1β), IL-6, and tumor necrosis factor-α (TNF-α), and oxidative markers, reactive oxygen species (ROS), and malondialdehyde (MDA), while elevating superoxide dismutase activity. Transcriptomics revealed SQJZ-mediated regulation of HIF-1. CHAC1 knockdown in vitro reduced LPS-induced apoptosis and inflammation, while HIF-1α activation reversed these effects. Additive suppression of inflammation was observed in NCM460 cells with combined CHAC1 knockdown and SQJZ treatment.

**Conclusion:**

SQJZ alleviates intestinal inflammation in CKD, potentially mediated by downregulation of CHAC1 and subsequent inactivation of the HIF-1 pathway, positioning SQJZ as a promising gut-targeted therapy in CKD.

## 1. Introduction

Chronic kidney disease (CKD), a global public health burden, is characterized by progressive renal dysfunction and systemic complications, which can advance to end-stage renal disease necessitating life-sustaining therapies, such as dialysis or renal transplantation [1, 2]. CKD affects more than 10% of the global population, contributing to elevated social burdens [2, 3]. While there are many identified contributors to CKD progression [4, 5], increasing evidence highlights the pivotal role of intestinal homeostasis imbalance as one of the drivers of systemic pathology, which underscores the crucial role of the gut-kidney axis in CKD [6, 7]. Intestinal inflammation, including aberrant cytokine secretion and elevated levels of proinflammatory and redox imbalance mediators, is an important manifestation of intestinal homeostasis imbalance, resulting in intestinal motility disorder [6]. Consequently, mitigating intestinal inflammation represents one of the critical therapeutic strategies for managing CKD.

Shengqing Jiangzhuo (SQJZ) capsule, a traditional Chinese herbal formulation, contains leaves of *Reynoutria japonica Houtt* (Huzhang), flowers of *Smilax glabra* Roxb. (Tufuling), flowers of *Styphnolobium japonicum* (L.) Schott (syn. *Sophora japonica* L.) (Huaihua), and roots of *Astralagus mongholicus* Bunge (Huangqi). Developed to treat chronic renal failure (CRF) via Traditional Chinese Medicine (TCM) principles, SQJZ aims to “strengthen healthy qi” (enhancing splenic and renal function), resolve turbidity, detoxify, and break blood stasis to promote tissue regeneration [8]. SQJZ has shown efficacy in delaying renal fibrosis in CRF across clinical, animal, and in vitro studies [8], and one of its main components, quercetin, can suppress renal fibrosis to ameliorate CRF [9]. Previous studies have also demonstrated that SQJZ capsule is able to alleviate diabetic nephropathy via activating the Kelch-like ECH-associated protein 1/nuclear factor erythroid-derived 2-related factor 2 pathway [10]. Building upon these documented renoprotective effects, we hypothesized that SQJZ capsule may also ameliorate CKD. Its specific roles in CKD, especially in intestinal inflammation, a critical pathological feature along the gut-kidney axis, and the underlying molecular pathways involving key regulatory genes, need to be further investigated.

CHAC glutathione-specific γ-glutamylcyclotransferase 1 (CHAC1), a member of the CHAC family of γ-glutamylcyclotransferases, is involved in different kinds of diseases [11], such as cerebral ischemia/reperfusion injury [12], alopecia [13], and cancers, including thyroid carcinoma [14], breast cancer [15], and lung adenocarcinoma [16]. Recently, CHAC1 has been found to be involved in some kidney-related diseases [17–19]. For example, Zhou and Zhang [17] have demonstrated that CHAC1 is upregulated in sepsis-induced acute kidney injury and it could stimulate oxidative stress to promote ferroptosis and apoptosis of human renal tubular epithelial cells. In diabetic kidney disease, the inhibition of CHAC1/nuclear factor kappa B can suppress inflammation in renal proximal tubular epithelial cells and repress the progression of diabetic kidney disease [18]. In addition, Dong et al. [19] have reported that CHAC1 deficiency can inhibit oxidative damage, enhance renal function, and reduce crystal deposition in calcium oxalate kidney stone formation. While CHAC1 has been predominantly studied in renal contexts, emerging evidence supports its relevance in intestinal physiology and pathology. A recent study on intestinal epithelial cells demonstrated that CHAC1 is endogenously expressed in intestinal epithelial cells and plays a functional role in regulating cellular responses to stress [20]. Specifically, this study revealed that heat stress-induced intestinal epithelial injury upregulates CHAC1 via the activating transcription factor 4 (ATF4)-C/EBP homologous protein (CHOP) signaling pathway. CHAC1, as a glutathione-specific γ-glutamylcyclotransferase, promotes glutathione degradation, leading to aggravated oxidative stress, mitochondrial dysfunction, and apoptosis in intestinal epithelial cells, key processes underlying intestinal barrier disruption and inflammation [20]. These findings indicate that CHAC1 is not only expressed in intestinal tissues but also actively involved in modulating oxidative stress and cell survival in gut epithelial cells. The researches above highlight CHAC1 as a potential regulator of inflammation, oxidative stress, and cellular damage in renal and potentially associated intestinal tissues, driving us to explore the role of CHAC1 in CKD and the association between CHAC1 and the SQJZ capsule.

The hypoxia-inducible factor (HIF) family, as a central mediator of hypoxia response, participates in maintaining renal homeostasis while also contributing to the development of various pathological conditions associated with CKD, such as anemia, inflammation, aberrant angiogenesis, and vascular calcification [21]. The HIF-1 signaling pathway was identified as the downstream regulatory pathway of SQJZ in this study. Our findings revealed the therapeutic efficacy of SQJZ capsule in alleviating CKD by inhibiting intestinal inflammation and elucidated its possible mechanistic involvement of CHAC1 and HIF-1 signaling, not only providing experimental evidence for SQJZ as a gut-targeted therapy for CKD but also suggesting CHAC1 as a potential therapeutic target for CKD-associated intestinal inflammation.

## 2. Materials and Methods

### 2.1. Preparation of Drugs

SQJZ capsules (0.5 g/capsule) were purchased from the First Affiliated Hospital of Guangzhou University of TCM. The powder of the SQJZ capsule was dissolved in normal saline to prepare a suspension with a concentration of 150 mg/mL. The dosage of SQJZ suspension administered to rats (0.8 mL/100 g/day) corresponds to 120 mg/kg/day, determined as follows: First, the clinical adult dose of SQJZ capsule is 9 g/day (0.5 g × 6 capsules × 3 times/day). Using the body surface area normalization method [22], the theoretical equivalent dose for rats is approximately 240 mg/kg/day. This calculation follows the formula: animal dose (mg/kg) = human dose (mg/kg) × (human *k*_m_ /animal *k*_m_), where the conversion factor (*k*_m_) is 0.16 for humans and 0.08 for rats. In addition, based on preliminary experiments, the reduced dose of 120 mg/kg/day was selected because it resulted in better animal status while retaining therapeutic efficacy. Adenine, obtained from Sigma–Aldrich (Darmstadt, Germany), was dissolved in normal saline to prepare a suspension with a concentration of 250 mg/mL.

### 2.2. Establishment of CKD Rat Model

A total of 30 specific pathogen-free male Sprague–Dawley rats (6–8 weeks old, 180–200 g), obtained from SPF Biotechnology Co., Ltd. (Beijing, China), were housed under a 12-h light/dark cycle with free access to water. All animal experiments were approved by the ethical approval of the First Affiliated Hospital of Guangzhou University of TCM (approval number: GZTCMF1-20230516).

To explore the effect of SQJZ capsule on CKD, after 1 week of acclimatization, 18 rats were randomly divided into three groups: Control (2 mL/day normal saline gavage for 8 weeks), CKD (0.8 mL/100 g/day adenine gavage for 4 weeks followed by 2 mL/day normal saline gavage for 4 weeks), and CKD + SQJZ (0.8 mL/100 g/day adenine gavage for 4 weeks followed by 0.8 mL/100 g/day SQJZ suspension gavage for 4 weeks) [23, 24]. Each group contained six rats. Group sizes were determined based published adenine-CKD models [25–27] and pre-experimental validation. Control and CKD groups received fixed-volume saline, while drug-treated groups received weight-adjusted doses (0.8 mL/100 g/day) to ensure precise compound delivery. On Day 55 (penultimate day of week 8), all rats were transferred to metabolic cages, and 24-h urine was collected into pre-chilled tubes containing 0.1% sodium azide. Urine samples were centrifuged (3000 × *g*, 10 min, 4°C) and supernatants were stored at −80°C for biochemical analysis. On Day 56, all rats were anesthetized through inhalation of isoflurane 6 h after the final drug/saline administration, and then euthanized by cervical dislocation. Blood specimens were harvested from the rats. Serum was separated (3,000 × *g*, 15 min, 4°C) and stored at −80°C. Kidney and colon tissues were excised, weighed, and documented photographically. Kidney tissues were divided into two aliquots: one subset was fixed in 4% paraformaldehyde for histopathological analysis, while the remaining portion was snap-frozen in liquid nitrogen for molecular evaluations. Colon tissues underwent identical fixation and cryopreservation protocols.

For the preparation of medicated serum, 12 rats were grouped as the SQJZ and negative control groups. Animals of the SQJZ group were administered intragastrically 0.8 mL/100 g/day SQJZ suspension for 4 days, while the rats from the negative control group were administered intragastrically 2 mL/100 g/day normal saline for 4 days [28]. After the final treatment for 1 h, rats were anesthetized through inhalation of isoflurane for collection of the abdominal aortic blood. Following centrifugation, serum samples were harvested, combined, and subjected to filtration using a 0.22 μm membrane. The prepared medicated serum was used for subsequent in vitro experiments with NCM460 cells to simulate the in vivo therapeutic effect of SQJZ in cellular models. Serum samples were pooled to reduce individual variability among rats, ensuring the obtained serum represented the average biological activity of SQJZ components in the circulation. The pooled samples then underwent heat inactivation at 56°C for 30 min before being cryopreserved at −80°C for subsequent analyses.

### 2.3. Histopathological Analysis via Hematoxylin-Eosin (HE) Staining

Renal and colon tissues were fixed in 4% paraformaldehyde, dehydrated through an ethanol gradient, cleared in xylene, and embedded in paraffin. Sections (4 μm) were stained with hematoxylin and eosin. Histopathological evaluation was performed by two independent researchers who were blinded to the experimental group assignments. Digital images of the stained sections were captured using a Nikon Eclipse Ni-U microscope equipped with a DS-Ri2 camera. All six animals per group (Control, CKD, and CKD + SQJZ) were included in histopathological assessments. For each animal, three nonconsecutive paraffin sections (separated by at least 4 μm) were analyzed. The scoring was performed manually by examining two nonoverlapping fields per section under 200× magnification. Discrepancies between observers were resolved by consensus. For renal tissues, semiquantitative pathological scoring was performed based on the following criteria: six parameters were assessed, including tubular atrophy, epithelial cell degeneration, epithelial cell necrosis, interstitial edema, renal interstitial fibrosis, and inflammatory cell infiltration. Each parameter was scored on a 0–3 scale: 0 = normal; 1 = mild damage; 2 = moderate damage; 3 = severe damage. The total score was the sum of individual scores (range: 0–18). Renal damage was classified as mild (total score ≤ 4), moderate (5–8), or severe (≥ 9). For colon tissues, pathological changes were evaluated using the Chiu's scoring system: 0 = normal mucosal villi; 1 = subepithelial space enlargement (typically at villus tips) with occasional capillary congestion; 2 = subepithelial space enlargement with moderate separation of the epithelial layer from the lamina propria; 3 = extensive separation of the epithelial layer from the lamina propria on both sides of villi, with partial villus tip damage; 4 = villus damage with exposure of lamina propria capillaries, possibly accompanied by increased cellular components in the lamina propria; 5 = lamina propria destruction, incompleteness, hemorrhage, or ulceration. Scores for each parameter were averaged across fields and sections to yield a single score per animal per parameter.

### 2.4. Transcriptomic Sequencing

Transcriptomic sequencing was performed on a total of eight samples, including two from the Control group, three from the CKD group, and three from the CKD + SQJZ group. For differential expression analysis, raw read counts of genes/transcripts were used as input data. For pairwise comparisons with biological replicates, DESeq2 was applied to calculate *p*-values, which were then adjusted using the Benjamini–Hochberg (BH) method to generate q-values, false discovery rate (FDR)-adjusted *p*-values. Differentially expressed genes (DEGs) were defined as those meeting the criteria: |log_2_FoldChange| >1 and q-value <0.05. For pairwise comparisons without biological replicates and multigroup comparisons, edgeR was used for analysis, with BH-adjusted q-values <0.05 as the threshold for statistical significance, while no fold change threshold was applied for multigroup analyses. To visualize the expression patterns of the DEGs, bidirectional hierarchical clustering analysis was conducted using the R package Pheatmap. The clustering was based on the expression levels of the same gene across different samples and the expression patterns of different genes within the same sample. The Euclidean distance metric was employed to calculate the distance matrix, and the complete linkage method was used for hierarchical clustering.

### 2.5. Bioinformatics Analysis

Following the identification of DEGs, Gene Ontology (GO) and Kyoto Encyclopedia of Genes and Genomes (KEGG) pathway enrichment analyses were performed using the clusterProfiler R package to elucidate the biological functions and pathways associated with the common DEGs among Control, CKD, and CKD + SQJZ groups. The enrichment results were filtered to select the most significant terms based on the smallest *p*-values. GO enrichment analysis results were visualized using bar plots and Circos plots, highlighting the most enriched terms in three categories: biological process, molecular function, and cellular component. The degree of enrichment was evaluated using the rich factor, FDR, and the number of genes enriched in each term. For KEGG pathway analysis, the top 20 most significantly enriched pathways (based on the smallest FDR values) were selected and visualized using bubble plots and Circos plots.

### 2.6. Cell Culture and Treatments

The human normal colon epithelial cell line NCM460, purchased from iCell Bioscience Inc. (Shanghai, China), was cultured in RPMI-1640 medium supplemented with 10% fetal bovine serum and 1% penicillin-streptomycin, and maintained in a humidified incubator at 37°C with 5% CO_2_. The culture medium was refreshed every 2–3 days, and cells were passaged or used for experiments when they reached 70%–80% confluence. To investigate the role of CHAC1, small interfering RNAs (siRNAs) targeting CHAC1 (si-CHAC1−1, si-CHAC1−2, and si-CHAC1−3) were used to knock down CHAC1 expression in NCM460 cells using Lipo6000. At the same time, a nontargeting siRNA (si-NC) was used as a negative control. The relative primer sequences were listed in Supporting Information 1: Table S1. Lipopolysaccharide (LPS) was used to induce inflammation. The concentration range of LPS was selected based on a previous study [29], which reported that 2–10 μg/mL LPS effectively induced inflammatory responses in similar cellular models. To further optimize the conditions for NCM460 cells, we expanded the range to 0, 5, 10, 15, and 20 μg/mL for 24 h, and additionally tested 10 μg/mL LPS at 0, 24, and 48 h to determine the optimal treatment time. Similarly, Fenbendazole-d3 (Fen-d3), the HIF-1 α agonist, was applied to activate the HIF-1 pathway. The concentration range of Fen-d3 was referenced from previous studies [30, 31], which reported effective activation of HIF-1 signaling using 1–20 μM Fen-d3 in various cell models. To determine the optimal concentration for NCM460 cells, we tested 0, 10, 15, 20, 25, and 30 μM for 24 h. Finally, NCM460 cells were divided into the following groups: Control (NCM460 cells without any treatment), LPS + si-NC (NCM460 cells infected with si-NC and treated with 10 μg/mL LPS), LPS + si-CHAC1 (NCM460 cells infected with si-CHAC1 and treated with 10 μg/mL LPS), LPS + si-CHAC1 + Fen-d3 (NCM460 cells infected with si-CHAC1 and treated with 10 μg/mL LPS and 25 μM Fen-d3) groups.

In the validation experiment of additive effect of SQJZ and CHAC1 knockdown, NCM460 cells were divided into Control (NCM460 cells cultured in medium containing 10% medicated serum from rats treated with saline), LPS + si-NC (NCM460 cells infected with si-NC, treated with 10 μg/mL LPS, and cultured in medium containing 10% medicated serum from rats treated with saline), LPS + si-CHAC1 (NCM460 cells infected with si-CHAC1, treated with 10 μg/mL LPS, and cultured in medium containing 10% medicated serum from rats treated with saline), LPS + SQJZ (NCM460 cells treated with 10 μg/mL LPS and cultured in medium containing 10% medicated serum from rats in SQJZ group), and LPS + SQZJ + si-CHAC1 (NCM460 cells infected with si-CHAC1, treated with 10 μg/mL LPS, and cultured in medium containing 10% medicated serum from rats in SQJZ group) groups.

### 2.7. Enzyme-Linked Immunosorbent Assay (ELISA)

The expression of blood urea nitrogen (BUN) and serum creatinine (SCR) in serum of rats, and urine albumin/creatinine ratio (ACR) from urine samples of rats were quantified using corresponding ELISA kits (Esebio, Shanghai, China) according to the manufacturer's protocol. The levels of interleukin-1β (IL-1β), IL-6, tumor necrosis factor-α (TNF-α), superoxide dismutase (SOD), reactive oxygen species (ROS), and malondialdehyde (MDA) in colon tissues or NCM460 cells were similarly assessed. For cytokine and oxidative stress marker measurements in colon tissues, approximately 100 mg of tissue was homogenized in 1 mL of ice-cold RIPA lysis buffer (Beyotime) containing 1% protease inhibitor cocktail. Homogenization was performed using a tissue homogenizer. The homogenates were then centrifuged at 12,000 × g for 15 min at 4°C. The resulting supernatants were collected, and the protein concentration was determined using a bicinchoninic acid (BCA) Protein Assay Kit (Thermo Fisher Scientific) according to the manufacturer's protocol. Samples were diluted with the homogenization buffer to equalize protein concentrations across samples before performing the ELISAs.

### 2.8. Real-Time Fluorescent Quantitative PCR (RT-qPCR)

Total RNA was isolated from NCM460 cells from experimental groups using TRIZOL reagent (Invitrogen, Carlsbad, CA, USA). RNA purity and concentration were determined by spectrophotometric analysis, with A260/A280 ratios between 1.9 and 2.0 confirming high-quality RNA. Reverse transcription was performed using a thermal cycler to generate cDNA. Quantitative PCR amplification was conducted with PowerTrack SYBR Green Master Mix kit (Thermo Fisher Scientific, Waltham, MA, USA), under the following cycling parameters: 95°C for 30 s (initial denaturation), followed by 40 cycles of 95°C for 10 s and 60°C for 30 s. GAPDH was selected as the endogenous reference gene for normalization of RT-qPCR data. The stability of GAPDH expression across the experimental conditions tested in this study was validated. Analysis of preliminary RT-qPCR data confirmed that GAPDH mRNA expression levels did not vary significantly (*p*  > 0.05) among the different treatment groups of NCM460 cells. Therefore, GAPDH was deemed suitable for use as a reference gene. Relative gene expression was calculated using the 2^−ΔΔCt^ method, with triplicate technical replicates per sample. Primer sequences were detailed in Supporting Information 1: Table S2.

### 2.9. Cell Counting Kit-8 (CCK-8) Assay

Cell viability was assessed through the CCK-8 assay according to the manufacturer's protocol. Briefly, NCM460 cells were seeded into 96-well plates at a density of 4 × 10^4^ cells/well and allowed to adhere overnight. After treatment with experimental agents, cells were washed twice, and incubated with 10% CCK-8 reagent (Beyotime, Shanghai, China) in maintenance medium for 2 h at 37°C. Absorbance at 450 nm was measured using a microplate reader. Data were normalized to untreated control groups to determine relative cell viability.

### 2.10. Western Blot

Total protein was extracted from cells or tissues using RIPA buffer (Beyotime) containing protease inhibitors. Protein concentrations were quantified via the BCA Assay kit (Thermo Fisher Scientific). Proteins were separated by 10% sodium dodecyl sulfate-polyacrylamide gel electrophoresis and transferred onto polyvinylidene fluoride membranes. Membranes were blocked with 5% non-fat milk for 2 h at room temperature and incubated overnight at 4°C with primary antibodies: anti-CHAC1 (1:1000, 15207–1-Ap, Proteintech, Wuhan, China), anti-HIF-1 α (1:1000, ab179483, Abcam, Cambridge, USA), anti-occludin (1:1000, ab216327, Abcam), anti-zona occludens 1 (ZO-1; 1:1000, ab221547, Abcam), anti-vascular endothelial growth factor (VEGF; 1:2000; AF1309; Beyotime), anti-glucose transporter 1 (GLUT1; 1:1000; AF1015; Beyotime), and anti-GAPDH (1:2000, ab181602, Abcam). After washing with TBST, membranes were incubated with secondary antibodies (ab288151; 1:5000; Abcam) for 1 h at room temperature. Protein bands were visualized using a Tanon 5200 chemiluminescence imaging system (Tanon, Shanghai, China) and quantified via ImageJ software. All raw gel images for Western blot results in this study were showed in the Supporting Information 2: Supporting File 1.

### 2.11. Apoptosis Detection by Flow Cytometry

Cell apoptosis was analyzed using an Annexin V-FITC/propidium iodide Apoptosis Detection Kit (Beyotime). Following treatment, NCM460 cells were harvested, washed twice with cold phosphate buffer solution, and resuspended in 195 μL binding buffer. Cells were stained with 5 μL Annexin V-FITC and 10 μL propidium iodide for 15 min in the dark at room temperature. Apoptotic rates were immediately quantified using a CytoFLEX flow cytometer (Beckman Coulter, Brea, CA, USA). Data were analyzed with CytExpert software.

### 2.12. Statistical Analysis

All the data were processed by GraphPad Prism 7.0 statistical software (GraphPad, San Diego, CA, USA), and the data were presented as multiple groups of repeated data or means ± standard deviation. The comparisons between two groups were analyzed by *t*-test, and those among multiple groups were analyzed by one-way ANOVA with Tukey's post hoc analysis. A *p*-value less than 0.05 was considered statistically significant.

## 3. Results

### 3.1. SQJZ Capsule Alleviates Renal Dysfunction in CKD Rats

**Figure 1:**
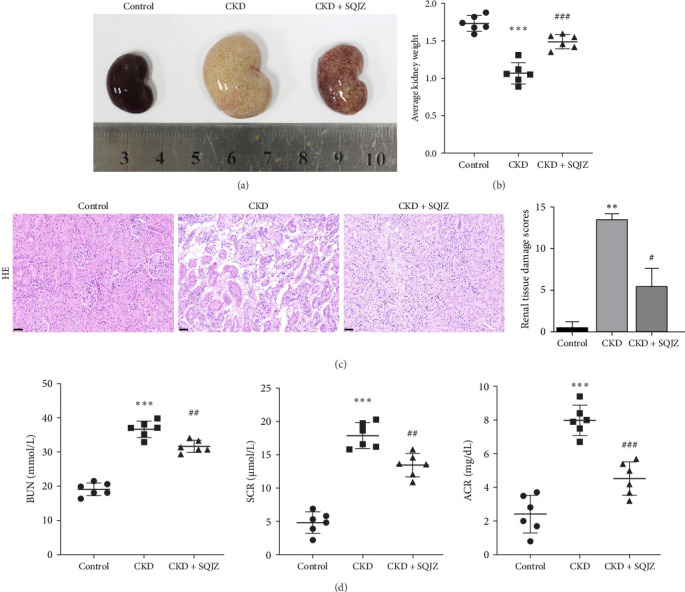
Shengqing Jiangzhuo (SQJZ) capsule alleviates renal function in chronic kidney disease (CKD) rats (*n* = 6). (A) Representative images of rat kidney tissues. (B) Average kidney weight of rats. (C) Hematoxylin and eosin (HE) staining of kidney tissues (magnification: 200×, scale bar: 50 μm) and semiquantitative renal damage scores. (D) Enzyme-linked immunosorbent assay (ELISA) results for blood urea nitrogen (BUN), serum creatinine (SCR), and urine albumin/creatinine ratio (ACR). *⁣*^*∗∗*^*p*  < 0.01, *⁣*^*∗∗∗*^*p*  < 0.001 vs. control group; ^#^*p*  < 0.05, ^##^*p*  < 0.01, ^###^*p*  < 0.001 vs. CKD group.

The kidneys of the Control group rats were bean-shaped, reddish-brown, firm, normal in size, with high gloss, and regular morphology (Figure 1A). In contrast, the CKD group exhibited significant “white kidney” phenomenon, with uneven surfaces and enlarged volumes compared to the Control group (Figure 1A). The CKD + SQJZ group showed improved glossiness, fewer irregularities, and a reddish color with white spots (Figure 1A). After weighing the kidney tissues, a lower weight was found in the CKD group compared to the Control group, while a higher weight was found in the CKD + SQJZ group relative to the CKD group (all *p*  < 0.001; Figure 1B). Histopathological analysis via HE staining indicated that the total renal damage score in the CKD group was significantly higher than that in the Control group (*p*  < 0.01; Figure 1C), indicating severe renal injury; while the score in the CKD + SQJZ group was markedly lower than that in the CKD group (*p*  < 0.05; Figure 1C), suggesting a significant alleviation of renal pathological damage (Figure 1C). The results of ELISA indicated that compared to the Control group, rats in the CKD group had significantly elevated BUN and SCR levels, and increased ACR level in urine (all *p*  < 0.001; Figure 1D). In contrast, lower BUN, SCR, and ACR were observed in the CKD + SQJZ group compared to the CKD group (*p*  < 0.01 or *p*  < 0.001; Figure 1D).

### 3.2. SQJZ Capsule Alleviates Intestinal Inflammation in CKD Rats

**Figure 2:**
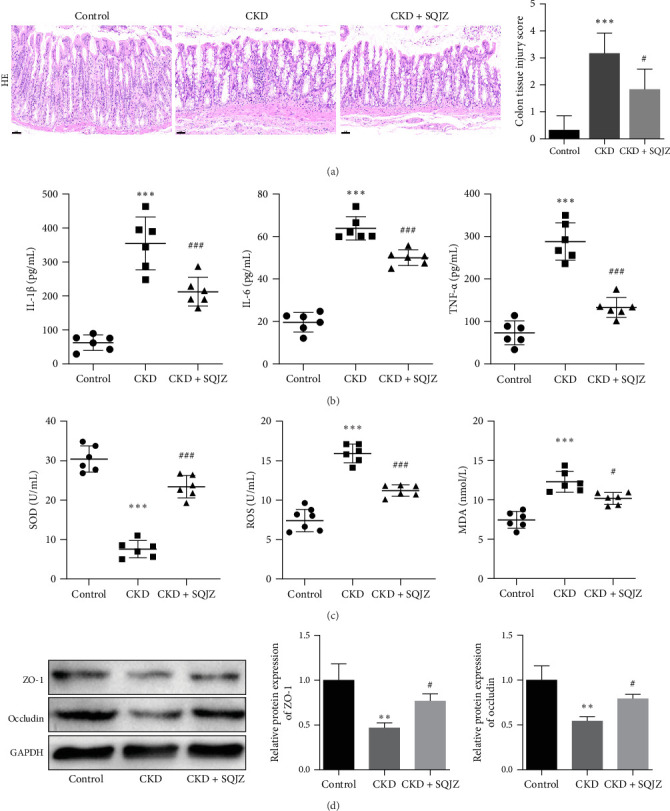
SQJZ capsule alleviates intestinal inflammation in CKD rats (*n* = 6). (A) HE staining of colon tissues (magnification: 200×, scale bar: 50 μm) and semi-quantitative colon mucosal damage scores. (B) ELISA results for interleukin-1β (IL-1β), IL-6, and tumor necrosis factor-α (TNF-α). (C) ELISA results for superoxide dismutase (SOD), reactive oxygen species (ROS), and malondialdehyde (MDA) in colon tissues. (D) The relative protein expression of occludin and zona occludens 1 (ZO-1) in colon tissues was detected by western blot. *⁣*^*∗∗∗*^*p*  < 0.001, *⁣*^*∗∗*^*p*  < 0.01 vs. Control group; ^#^*p*  < 0.05, ^###^*p*  < 0.001 vs. CKD group.

Colon tissues from rats of the CKD group displayed thickened walls, mononuclear leukocyte infiltration, and disrupted villi architecture, whereas SQJZ treatment alleviated these pathological changes (Figure 2A). Semiquantitative assessment using Chiu's scoring system further confirmed the improvement. The colon tissue damage score in the CKD group was significantly higher than that in the Control group (*p*  < 0.001; Figure 2A), indicating obvious mucosal injury; while the score in the CKD + SQJZ group was markedly lower than that in the CKD group (*p*  < 0.05; Figure 2A), suggesting a significant reduction in intestinal pathological damage. Compared with the CKD group, reduced levels of proinflammatory cytokines, IL-1β, IL-6, and TNF-α, and oxidative stress markers, ROS and MDA were determined in the CKD + SQJZ group (*p*  < 0.001 or *p*  < 0.05; Figure 2B,C), which were significantly elevated in the CKD group relative to the Control group (all *p*  < 0.001, Figure 2B,C). Conversely, SOD activity, a key antioxidant enzyme suppressed by CKD, was restored post-treatment (*p*  < 0.001; Figure 2C). Western blot demonstrated that SQJZ upregulated the expression level of tight junction proteins, occludin, and ZO-1, which were inhibited by CKD, suggesting a potential improvement in intestinal barrier integrity (*p*  < 0.05 or *p*  < 0.01; Figure 2D).

### 3.3. Transcriptomic Insights Into SQJZ-Mediated Anti-Inflammatory Pathways

**Figure 3:**
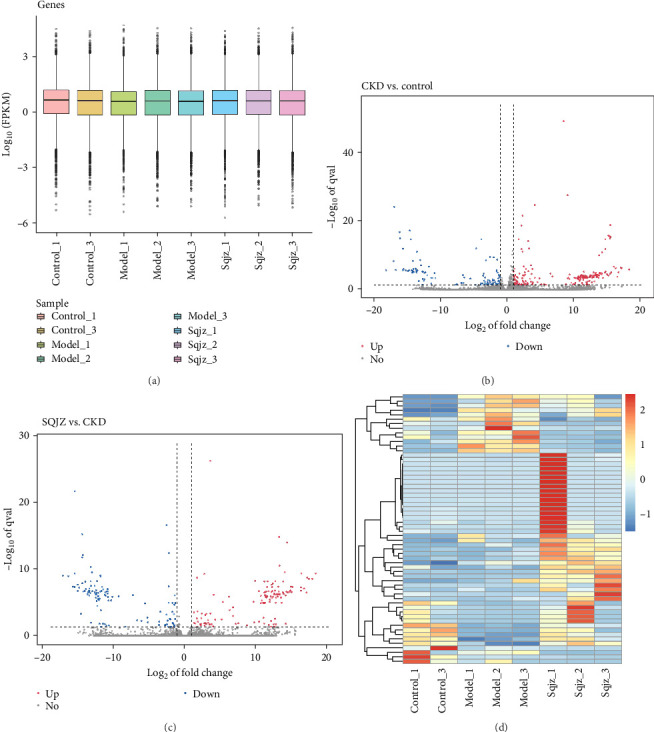
Identification of differentially expressed genes (DEGs). (A) A boxplot of gene expression in Control, CKD, and CKD + SQJZ groups. (B) A volcano plot of DEGs between the CKD and the Control group. Red points presented upregulated DEGs, bule points presented downregulated DEGs, and gray dots indicated no significant difference in expression levels. (C) A volcano plot of DEGs between the CKD and the CKD + SQJZ group. Red points presented upregulated DEGs, bule points presented downregulated DEGs, and gray dots indicated no significant difference in expression levels. (D) A heatmap of gene expression across three groups.

**Figure 4:**
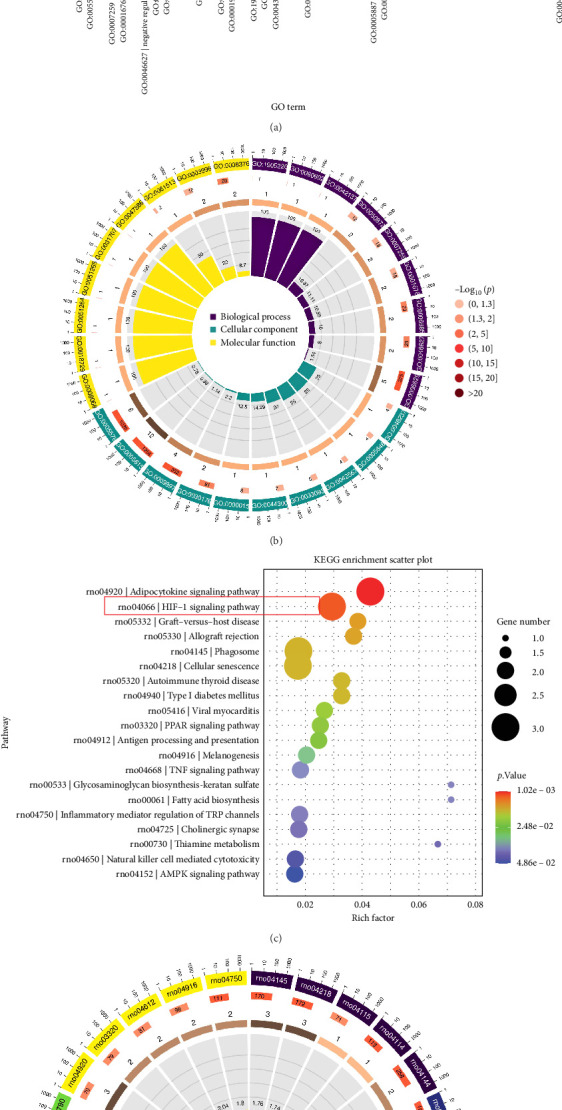
Functional and pathway enrichment analysis of common DEGs among Control, CKD, and CKD + SQJZ groups. The results of the Gene Ontology (GO) enrichment analysis of DEGs showed in (A) a bar chart and (B) a Circos plot. The results of the Kyoto Encyclopedia of Genes and Genomes (KEGG) pathway analysis showed in (C) a bubble chart and (D) a Circos plot.

For transcriptome profiling, colon samples were collected from the Control group (*n* = 2), CKD group (*n* = 3), and CKD + SQJZ group (*n* = 3). To ensure data reliability, normalization, and cross-comparability were evaluated across all groups. The boxplots revealed that most samples clustered centrally within their respective groups, conforming to expected distributions (Figure 3A). RNA sequencing of colon tissues identified 71 upregulated and 28 downregulated genes between the CKD group and the Control group, with 58 upregulated and 41 downregulated genes between the CKD group and the CKD + SQJZ group (Figure 3B,C). Bidirectional clustering analysis highlighted distinct gene expression profiles across groups (Figure 3D). GO enrichment analysis of common DEGs among Control, CKD, and CKD + SQJZ groups revealed significant associations with lipid metabolic process, membrane, and protein binding (Figure 4A,B). KEGG pathway analysis of common DEGs among three groups identified the HIF-1 signaling, the adipocytokine signaling, and the peroxisome proliferators-activated receptor (PPAR) pathways as key contributors to SQJZ efficacy (Figure 4C,D; Supporting Information 1: Table S3). The HIF-1 signaling pathway was prioritized for further investigation due to well-established roles in mediating intestinal inflammation and oxidative stress. The information of three DEGs, endothelin 1 (Edn1), enolase 3 (Eno3), and calcium/calmodulin-dependent protein kinase II alpha (Camk2a), that were enriched in the HIF-1 signaling pathway was listed in Supporting Information 1: Table S4.

### 3.4. SQJZ Capsule May Ameliorate CKD-Induced Intestinal Inflammation via CHAC1 Inhibition and HIF-1 Pathway Regulation

**Figure 5:**
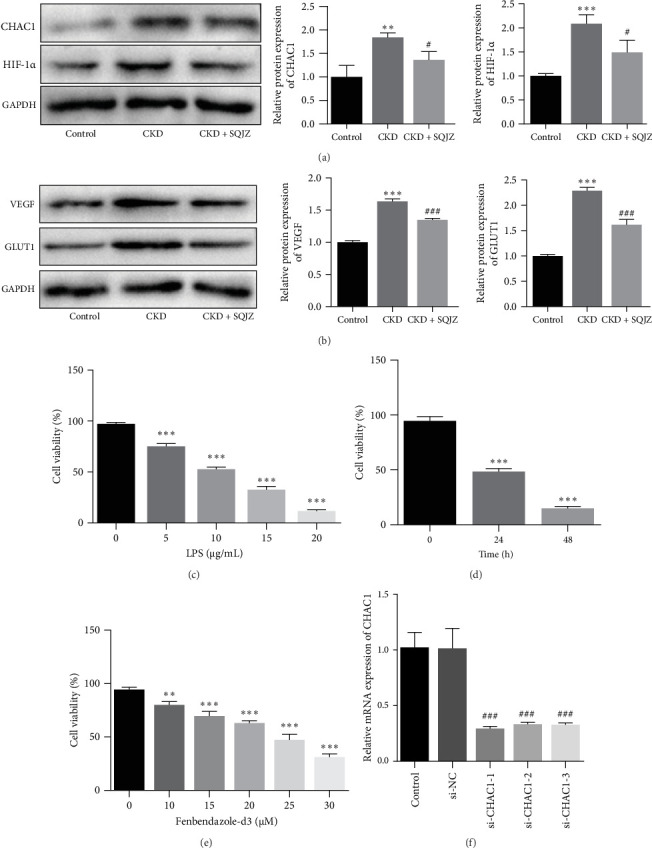
Identification of the optimal treatment conditions. (A, B) The protein expression of CHAC glutathione-specific γ-glutamylcyclotransferase 1 (CHAC1), hypoxia-inducible factor 1 α (HIF-1 α), vascular endothelial growth factor (VEGF), and glucose transporter 1 (GLUT1) in colon tissues was detected by western blot; *⁣*^*∗∗∗*^*p*  < 0.001, *⁣*^*∗∗*^*p*  < 0.01 vs. Control group; ^###^*p*  < 0.001, ^#^*p*  < 0.05 vs. CKD group. (C) Cell counting kit-8 (CCK-8) assay was used to measure the viability of NCM460 cells after 24 h of intervention with lipopolysaccharide (LPS) at concentrations of 0, 5, 10, 15, and 20 μg/mL; *⁣*^*∗∗∗*^*p*  < 0.001 vs. 0 μg/mL. (D) CCK-8 assay of NCM460 cell viability after 10 μg/mL LPS treatment for 0, 24, and 48 h; *⁣*^*∗∗∗*^*p*  < 0.001 vs. 0 h. (E) CCK-8 assay was used to measure the viability of NCM460 cell viability after 24 h of intervention with Fenbendazole-d3 (Fen-d3) at concentrations of 0, 10, 15, 20, 25, and 30 μM; *⁣*^*∗∗∗*^*p*  < 0.001, *⁣*^*∗∗*^*p* < 0.01 vs. 0 μM. (F) The CHAC1 knockdown efficiency was detected by real-time fluorescent quantitative PCR; ^###^*p*  < 0.001 vs. si-NC group.

Western blot analysis revealed significantly elevated expression of CHAC1 and the HIF-1 regulatory subunit HIF-1 α in the colon tissues of CKD rats compared to the Control group (*p*  < 0.01 *or* *p*  < 0.001; Figure 5A). However, relative to the CKD group, SQJZ treatment markedly reduced their expression levels (*p*  < 0.05; Figure 5A), suggesting CHAC1 and HIF-1 α may serve as critical targets for SQJZ to exert its therapeutic effects. To functionally corroborate the transcriptomic prediction of HIF-1 pathway involvement, we examined protein expression of two canonical HIF-1 transcriptional targets, VEGF, and GLUT1, in colon tissues. Western blot analysis revealed that compared to the Control group, CKD rats exhibited significantly elevated VEGF (*p*  < 0.001) and GLUT1 (*p*  < 0.001) expression (Figure 5B). SQJZ treatment reversed these changes, reducing VEGF (*p*  < 0.001) and GLUT1 (*p*  < 0.001) levels, confirming functional suppression of HIF-1 signaling activity. These findings confirmed that SQJZ suppressed pathological HIF-1 pathway activation in CKD-associated intestinal inflammation.

To evaluate the impact of CHAC1 on intestinal inflammation, LPS-induced inflammatory models were established using NCM460 cells. CCK-8 assays demonstrated that 10 μg/mL of LPS reduced cell viability to approximately 50% after 24 h, which was selected for subsequent experiments (Figure 5C,D). Similarly, to further elucidate the role of HIF-1 α in the therapeutic effects of SQJZ on intestinal inflammation, the HIF-1 α agonist Fen-d3 was employed. The results of CCK-8 demonstrated a gradual decrease in cell viability with increasing Fen-d3 concentrations (all *p*  < 0.001; Figure 5E), with cell viability of approximately 50% at 25 μM Fen-d3. RT-qPCR confirmed efficient CHAC1 knockdown in NCM460 cells using si-CHAC1-1, si-CHAC1-2, and si-CHAC1-3 (all *p*  < 0.001; Figure 5F), and si-CHAC1-1 was selected to apply for subsequent experiments, which exhibited superior silencing efficacy compared to si-CHAC1-2 and si-CHAC1-3.

**Figure 6:**
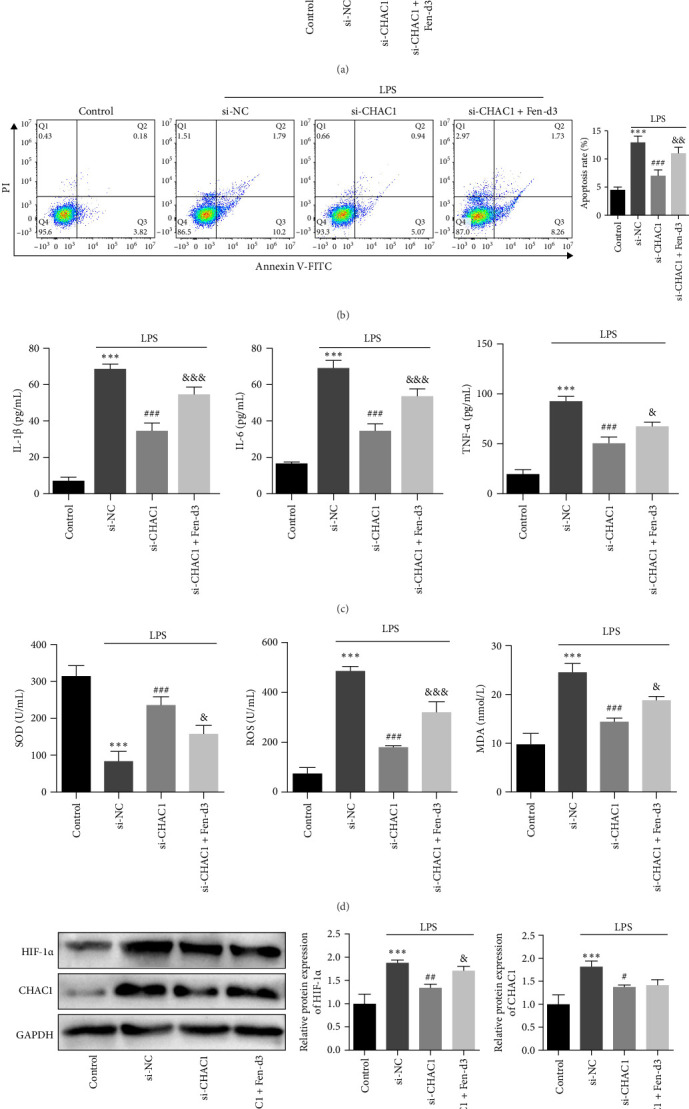
SQJZ capsule may ameliorate CKD-induced intestinal inflammation via CHAC1 inhibition and HIF-1 pathway regulation. (A) CCK-8 assay was used to measure the viability of NCM460 cell viability in different groups. (B) Flow cytometry was used to detect cell apoptosis in each group. (C) ELISA was used to detect the expression of IL-1β, IL-6, and TNF-α in each group. (D) ELISA was used to detect the expression of SOD, ROS, and MDA in each group. (E) The expression of HIF-1 α and CHAC1 in each group of cells was detected by western blot. *⁣*^*∗∗∗*^*p*  < 0.001 vs. Control group; ^###^*p*  < 0.001, ^##^*p*  < 0.01, ^#^*p*  < 0.05 vs. LPS + si-NC group; ^&&&^*p*  < 0.001, ^&&^*p*  < 0.01, ^&^*p*  < 0.05 vs. LPS + si-CHAC1 group.

CCK-8 assays indicated that CHAC1 knockdown significantly improved cell viability compared to the si-NC + LPS group (*p*  < 0.001; Figure 6A), while HIF-1 activation reversed this protective effect (*p*  < 0.001, Figure 6A). Flow cytometry revealed reduced apoptosis in the LPS + si-CHAC1 group relative to the si-NC + LPS group (*p*  < 0.001; Figure 6B), which was counteracted by Fen-d3 treatment (*p*  < 0.01, Figure 6B). ELISA results demonstrated that CHAC1 silencing suppressed LPS-induced upregulation of pro-inflammatory cytokines, IL-1β, IL-6, and TNF-α, and oxidative stress markers, ROS and MDA, while enhancing antioxidant SOD activity (all *p*  < 0.001, Figure 6C,D). Conversely, HIF-1 α activation by Fen-d3 antagonized these beneficial effects based on the results of elevated IL-1β, IL-6, TNF-α, ROS, and MDA and reduced SOD activity (*p*  < 0.001 or *p*  < 0.05; Figure 6C,D).

Western blot analysis further confirmed that CHAC1 knockdown downregulated HIF-1 α expression in NCM460 cells, which was restored by Fen-d3 (*p*  < 0.01 or *p*  < 0.05; Figure 6E). However, the application of Fen-d3 displayed no influence on CHAC1 expression, suggesting the HIF-1 α may be located downstream of CHAC1 and exert its functional role under the regulation of CHAC1.

### 3.5. Additive Effects of CHAC1 Knockdown and SQJZ Treatment on Alleviating Intestinal Inflammation

**Figure 7:**
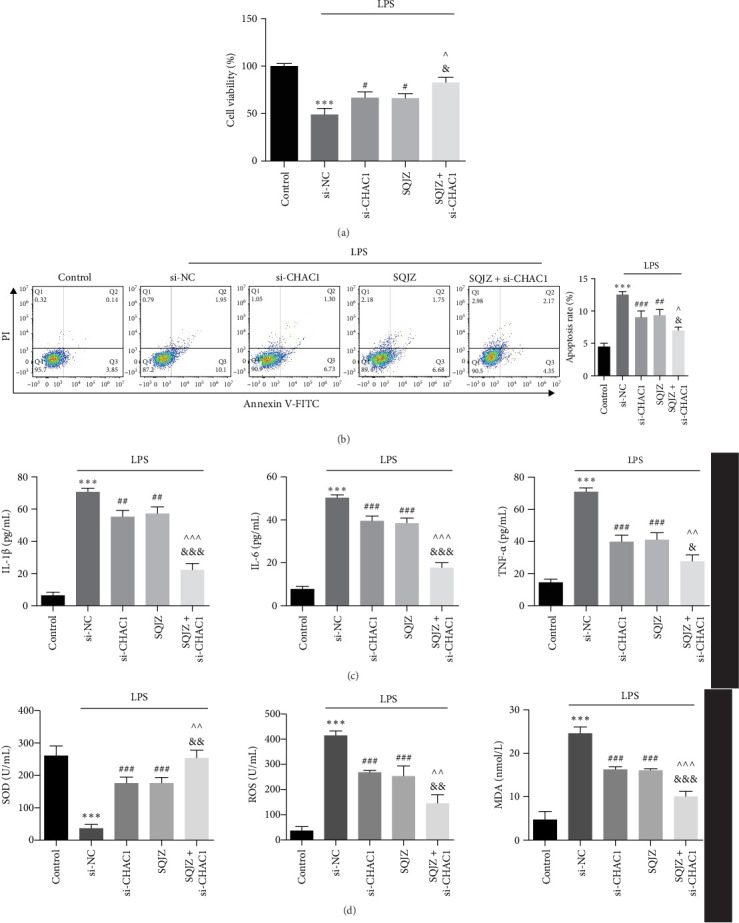
Additive effects of CHAC1 knockdown and SQJZ treatment on alleviating intestinal inflammation. (A) CCK-8 assay was used to measure the viability of NCM460 cell viability in different groups. (B) Flow cytometry was used to detect cell apoptosis in each group. (C) ELISA was used to detect the expression of IL-1β, IL-6, and TNF-α in each group. (D) ELISA was used to detect the expression of SOD, ROS, and MDA in each group. *⁣*^*∗∗∗*^*p*  < 0.001 vs. Control group; ^###^*p*  < 0.001, ^##^*p*  < 0.01, ^#^*p*  < 0.05 vs. LPS + si-NC group; ^&&&^*p*  < 0.001, ^&&^*p*  < 0.01, ^&^*p*  < 0.05 vs. LPS + si-CHAC1 group; ^^^^^*p*  < 0.001, ^^^^*p*  < 0.01, ^^^*p*  < 0.05 vs. LPS + SQJZ group.

CCK-8 assays showed that both CHAC1 knockdown and SQJZ treatment independently enhanced cell viability compared to the si-NC group, with the SQJZ + si-CHAC1 group exhibiting the highest viability (all *p*  < 0.05; Figure 7A). Flow cytometry revealed that SQJZ + si-CHAC1 collectively enhanced apoptosis reduction compared to the si-CHAC1 and SQJZ groups (*p*  < 0.05; Figure 7B). Similarly, ELISA results indicated that the combination of CHAC1 knockdown and SQJZ treatment more effectively suppressed inflammatory cytokines, IL-1β, IL-6, and TNF-α, and oxidative stress, ROS and MDA, while elevating SOD levels compared to the si-CHAC1 and SQJZ groups (*p*  < 0.001 or *p*  < 0.01 or *p*  < 0.05; Figure 7C,D). These results demonstrated that knockdown of CHAC1 could enhance the improvement effect of SQJZ capsule on intestinal inflammation, suggesting CHAC1 may be the target of SQJZ capsule in CKD-associated intestinal inflammation.

## 4. Discussion

CKD, a multifaceted disorder characterized by renal dysfunction and systemic complications, imposes significant global health and economic burdens due to its high prevalence and limited therapeutic options [32]. The gut-kidney axis plays a pivotal role in the CKD progression, highlighting the intestine as both a source and amplifier of CKD-related complications and positioning intestinal inflammation as one of the therapeutic targets [33, 34]. In this study, we demonstrated that the traditional Chinese herbal formulation SQJZ capsule not only could improve renal function but also suppress intestinal inflammation in CKD, with CHAC1 inhibition and HIF-1 α pathway regulation as key mechanistic drivers, displaying the therapeutic potential of gut-centric interventions in CKD management.

The rodent model of CKD induced by adenine administration is a widely accepted preclinical system for evaluating renal functional decline and therapeutic efficacy [24, 35]. It faithfully recapitulates histopathological hallmarks and disease progression analogous to human CKD [24]. Administration of adenine leads to noticeable pathological alterations, including renal hypertrophy and structural abnormalities [24, 35]. Our in vivo experiments demonstrated that SQJZ treatment significantly attenuated renal dysfunction in adenine-induced CKD rats, as evidenced by reduced serum BUN, SCR, and urinary ACR levels, and histopathological improvements, including diminished mesangial hyperplasia and inflammatory infiltration, which corroborated the renoprotective effects of SQJZ capsule.

CKD progression is accompanied by severe intestinal inflammation, which contributes to intestinal dysmotility with common clinical features including abdominal distension, bloating, and episodes of nausea accompanied by vomiting [36]. Owing to diminished intestinal peristalsis, patients often encounter challenges with enteral nutrition delivery, which may precipitate secondary complications such as microbial translocation across the gut barrier and protein-energy wasting [37, 38]. In this research, administration of adenine induced colon tissue damage and elevated inflammation levels of the colon, aligning with prior studies. However, SQJZ administration suppressed intestinal inflammation, suggesting SQJZ capsules could alleviate CKD by inhibiting intestinal inflammation to some extent. Impaired intestinal barrier function is a recognized hallmark of CKD pathophysiology, contributing to systemic inflammation through increased permeability to microbial products [39]. In this study, SQJZ treatment significantly upregulated the expression of occludin and ZO-1, two critical tight junction proteins that regulate the paracellular “leak pathway” for medium-sized solutes (10–20 kDa) [39]. Their downregulation in CKD directly associates with barrier dysfunction and precedes endotoxin translocation in preclinical models [40, 41]. Thus, the restoration of occludin and ZO-1 expression by SQJZ suggests a potential mechanism for ameliorating intestinal barrier integrity in CKD, although functional permeability assays would be required to definitively confirm improved barrier integrity.

CHAC1 has recently emerged as a mediator of inflammation. For example, Perra et al. [42] have indicated that CHAC1 modulates inflammatory responses in bronchial epithelial cells following *Pseudomonas aeruginosa* infection, potentially contributing to the persistent hyperinflammation observed in the airways of cystic fibrosis patients. The inhibition of CHAC1 is involved in suppressing the inflammatory response of renal proximal tubular epithelial cells and alleviating diabetic kidney disease [18]. Li et al. [43] have reported that caffeic acid attenuates ferroptosis-mediated inflammatory responses in psoriatic keratinocytes by suppressing the activation of CHAC1. In this research, SQJZ suppressed the expression of CHAC1 in the colon tissues of CKD rats, and knockdown of CHAC1 inhibited the inflammatory response of LPS-induced NCM460 cells. In addition, CHAC1 knockdown and SQJZ treatment exerted a significant additive effect on the inhibition of inflammation. The above results suggest that CHAC1 may be the target of SQJZ to inhibit intestinal inflammation and alleviate CKD.

The HIF-1 signaling pathway, identified as the downstream regulatory pathway of SQJZ in this study, serves as an important contributor to inflammation. HIF-1 is consisting of two dissimilar part, HIF-1 α and HIF-1β, each subunit has own specific role [44]. As the oxygen-sensitive subunit, HIF-1 α protein expression and stability is the primary driver of HIF-1 transcriptional activity [45]. Zhao et al. [46] have found that suppression of HIF-1 α alleviated inflammatory responses and redox imbalance in vascular cells subjected to hyperglycemia and hypoxia-induced damage. Phenothiazine modulates NLRP3 inflammasome-related inflammation through the HIF-1 α signaling pathways, contributing to neuroprotection in both hypothermic and normothermic conditions following acute ischemic stroke [47]. In CKD, downregulated HIF-1 α has been found to inhibit the vascular calcification and glycolysis of CKD mice [48]. Zhao et al. [49] have reported that HIF-1 α stimulates the Notch-1 signal to promote renal fibrosis in cisplatin-induced CKD mice. In this study, we found that SQJZ reduced HIF-1 α protein expression and downstream HIF-1 transcriptional targets, VEGF and GLUT1, collectively indicating suppression of HIF-1 signaling at the molecular level, and the treatment of HIF-1 α agonist Fen-d3 reversed the effect of CHAC1 knockdown on LPS-induced NCM460 cells. In addition, the expression of HIF-1 α was affected by CHAC1 level, but the Fen-d3 treatment had no effect on CHAC1. The above results suggest that CHAC1 knockdown may inhibit intestinal inflammation in CKD suppressing HIF-1 α protein accumulation, thereby inactivating the HIF-1 signaling pathway.

The coordinated downregulation of CHAC1 and HIF-1 α by SQJZ likely stems from its multicomponent phytochemistry. Quercetin, a key flavonoid from *Sophora japonica L*. (Huaihua), directly binds the PAS-B domain of HIF-1 α to inhibit dimerization and transcriptional activity, as demonstrated in rheumatoid arthritis synoviocytes [50]. This aligns with our observation of HIF-1 α suppression in colon tissues. Furthermore, *Reynoutria japonica* (Huzhang), another SQJZ component, contains resveratrol and emodin. Computational studies confirm that resveratrol binds HIF-1 α with high affinity (–5.5 kcal/mol) through hydrophobic interactions, destabilizing its structure and inhibiting hypoxic signaling in pancreatic cancer [51]. Emodin has been demonstrated to promote the Sirtuin 6-mediated deacetylation of HIF-1 α by reducing ERα levels, which otherwise inhibits this deacetylation process [52]. SQJZ may similarly leverage polyphenols to disrupt HIF-1 α stability. For CHAC1, SQJZ components may act indirectly through upstream signaling pathways. The ATF4-CHOP pathway is a key regulator of CHAC1 transcription [20]. Astragaloside IV, a major saponin from *Astragalus mongholicus* (Huangqi), could inhibit the ATF4-CHOP pathway to ameliorates podocyte apoptosis [53, 54]. Resveratrol was found to inhibit intestinal aging by downregulating ATF4-CHOP signaling pathway [55]. In cerebral ischemia and reperfusion injury, quercetin suppressed ATF4 and CHOP expression to reduce endoplasmic reticulum stress [56]. These findings collectively suggest that the main components of SQJZ may synergistically inhibit the ATF4-CHOP signaling pathway, thereby reducing the transcriptional activation of CHAC1. The additive reduction in inflammation with combined CHAC1 knockdown and SQJZ treatment supports multi-target modulation of the CHAC1/HIF-1 α axis. Future studies should validate direct binding interactions between SQJZ phytochemicals and CHAC1/HIF-1 α.

This study has several limitations. First, the transcriptomic analysis utilized a small Control group (*n* = 2) due to budget constraints, which may reduce statistical power for detecting subtle gene expression changes. While the FDR-controlled screening identified candidate pathways for validation, larger cohorts are needed in future studies to ensure transcriptomic robustness. Second, the 4-day administration for medicated serum preparation in this study, while shorter than the 4-week in vivo treatment, aligns with common practices in TCM research. Multiple studies have demonstrated that short-term dosing (3–7 days) for serum collection is sufficient to capture the bioactive profile of herbal formulas, even when in vivo interventions extend for weeks to months [57–59]. Importantly, our data validate the biological relevance of this approach: the suppression of proinflammatory cytokines (IL-1β and IL-6) and oxidative stress markers (ROS) by SQJZ-containing serum in vitro mirrored the in vivo effects observed after 4-week treatment. Such consistency supports that short-term medicated serum retains the core anti-inflammatory properties of SQJZ, making it a suitable tool for preliminary mechanistic exploration. However, we acknowledge that long-term in vivo administration may involve metabolic adaptations or cumulative effects not fully captured by short-term serum. Future studies will employ metabolomic profiling to compare the component profiles of acute vs. chronic serum, aiming to refine the translational relevance of in vitro models. Third, although this work identifies CHAC1/HIF-1 α signaling as a potential pathway for SQJZ's anti-inflammatory effects, certain mechanistic aspects require further validation. Specifically, the functional relationship between CHAC1 and HIF-1 α was established through genetic and pharmacological perturbations, but direct evidence of SQJZ components binding to CHAC1 is lacking. Future studies should employ molecular docking simulations, co-immunoprecipitation assays, or drug affinity responsive target stability (DARTS) techniques to verify physical interactions. Additionally, tissue-specific CHAC1 knockout models would help confirm its role as a primary therapeutic target in vivo. Fourth, although our data demonstrate that SQJZ treatment upregulates expression of tight junction proteins (occludin and ZO-1) in colon tissues, molecular indicators commonly associated with enhanced barrier function, we recognize that definitive evidence of improved intestinal barrier integrity requires functional validation through methods, such as FITC-dextran permeability assays or transepithelial electrical resistance measurements. The absence of these functional tests limits our ability to conclusively state that structural protein changes translate to physiological barrier restoration. Future investigations should directly assess barrier permeability in vivo and in vitro to establish functional correlates of the observed molecular improvements.

In conclusion, this study identified the CHAC1/HIF-1 α axis as a novel mechanistic target for SQJZ in alleviating CKD-associated intestinal inflammation. SQJZ may alleviate intestinal inflammation in CKD, potentially mediated by downregulation of CHAC1 and subsequent inactivation of the HIF-1 α pathway. These results not only advance our understanding of gut-kidney crosstalk in CKD but also position SQJZ as a promising gut-centric therapy.

## Data Availability

The datasets used and/or analyzed during the current study are available from the corresponding author on reasonable request.

## Supporting Information

Supporting Information 1 Table S1. The primer sequences of siRNAs. Table S2. The primer sequences for RT-qPCR Table S3. The top 10 KEGG pathways enriched by common DEGs among control, CKD, and CDK + SQJZ. Table S4. DEGs enriched with the HIF-1 signaling pathway in transcriptome analysis.

Supporting Information 2 Supporting File 1. Raw gel images for western blot results.
