# Supplementary material for: Shengqing Jiangzhuo Capsule Alleviates Intestinal Inflammation in Chronic Kidney Disease by Downregulating CHAC1 to Inactivate the HIF-1 Pathway
Source: Mediators Inflamm. 2025 Nov 6;2025:2173234. doi: 10.1155/mi/2173234 (PMC12615044; doi:10.1155/mi/2173234)
Supplement: Supporting Information 1 — Table S1. The primer sequences of siRNAs. Table S2. The primer sequences for RT-qPCR Table S3. The top 10 KEGG pathways enriched by common DEGs among control, CKD, and CDK + SQJZ. Table S4. DEGs enriched with the HIF-1 signaling pathway in transcriptome analysis. [file 2173234.f1.docx]

**Table S1. The primer sequences of siRNAs**

| Name | Sequences（5’-3’） |
| --- | --- |
| si-NC-F | AAGUUUCAAGUAGUAAGUCAG |
| si-NC-R | GACUUACUACUUGAAACUUUA |
| si-CHAC1-F-1 | UAAAGUUUCAAGUAGUAAGUC |
| si-CHAC1-R-1 | CUUACUACUUGAAACUUUAUU |
| si-CHAC1-F-2 | UAAAUAAAGUUUCAAGUAGUA |
| si-CHAC1-R-2 | CUACUUGAAACUUUAUUUAUU |
| si-CHAC1-F-3 | UUUUAUGGGUAACAGUAUGGG |
| si-CHAC1-R-3 | CAUACUGUUACCCAUAAAACU |

**Table S2. The primer sequences for RT-qPCR**

| Name | Sequences（5’-3’） |
| --- | --- |
| H-GAPDH-F | AACGGATTTGGTCGTATTGGGCGC |
| H-GAPDH-R | TCCTGGAAGATGGTGATGGGATTT |
| H-CHAC1-F | CGAACACCCCGCCCACCTCGCAGT |
| H-CHAC1-R | CCAGGGAGCCGTACCCGAAAATCC |

**Table S3. The top 10 KEGG pathways enriched by common DEGs among Control, CKD, and CDK + SQJZ.**

| Pathway ID | Term | Pvalue | Rich.Factor | Q.value |
| --- | --- | --- | --- | --- |
| [rno04920](https://www.kegg.jp/entry/rno04920) | Adipocytokine signaling pathway | 0.001024498 | 0.0429 | 0.095278279 |
| [rno04066](https://www.kegg.jp/entry/rno04066) | HIF-1 signaling pathway | 0.00302442 | 0.0294 | 0.140635547 |
| [rno05332](https://www.kegg.jp/entry/rno05332) | Graft-versus-host disease | 0.009731728 | 0.0385 | 0.153682912 |
| [rno05330](https://www.kegg.jp/entry/rno05330) | Allograft rejection | 0.010465496 | 0.037 | 0.153682912 |
| [rno04145](https://www.kegg.jp/entry/rno04145) | Phagosome | 0.012460091 | 0.0176 | 0.153682912 |
| [rno04218](https://www.kegg.jp/entry/rno04218) | Cellular senescence | 0.01285908 | 0.0174 | 0.153682912 |
| [rno04940](https://www.kegg.jp/entry/rno04940) | Type I diabetes mellitus | 0.013220035 | 0.0328 | 0.153682912 |
| [rno05320](https://www.kegg.jp/entry/rno05320) | Autoimmune thyroid disease | 0.013220035 | 0.0328 | 0.153682912 |
| [rno05416](https://www.kegg.jp/entry/rno05416) | Viral myocarditis | 0.019560643 | 0.0267 | 0.191070132 |
| [rno03320](https://www.kegg.jp/entry/rno03320) | PPAR signaling pathway | 0.021566003 | 0.0253 | 0.191070132 |

**Table S4. DEGs enriched with the HIF-1 signaling pathway in transcriptome analysis**

| Gene_ID | Gene_name | Full name | Pvalue |
| --- | --- | --- | --- |
| ENSRNOG00000014361 | Edn1 | endothelin 1 | 1.36e-06 |
| ENSRNOG00000018712 | Camk2a | calcium/calmodulin-dependent protein kinase II alpha | 7.83e-09 |
| ENSRNOT00000005612 | Eno3 | enolase 3 | 1.05e-4 |
