# Supplementary material for: Shengqing Jiangzhuo Capsule Alleviates Intestinal Inflammation in Chronic Kidney Disease by Downregulating CHAC1 to Inactivate the HIF-1 Pathway
Source: Mediators Inflamm. 2025 Nov 6;2025:2173234. doi: 10.1155/mi/2173234 (PMC12615044; doi:10.1155/mi/2173234)
Supplement: Supporting Information 2 — Supporting File 1. Raw gel images for western blot results. [file 2173234.f2.pdf]

Figure 2D ZO-1

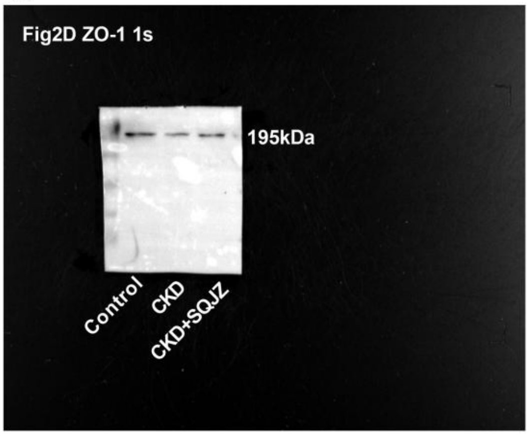

Representative blot

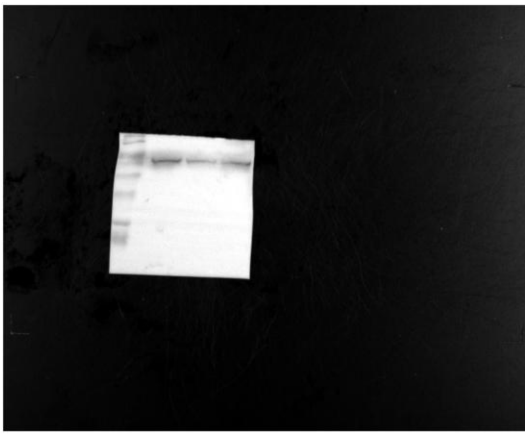

Biological replicate 2

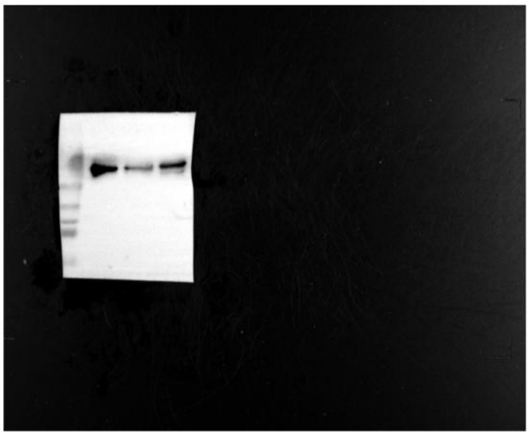

Biological replicate 3

Figure 2D GAPDH

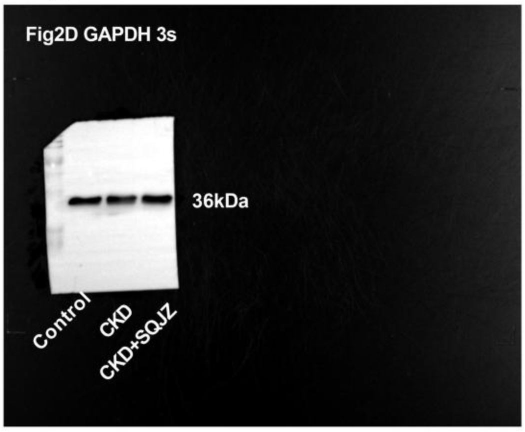

Representative blot

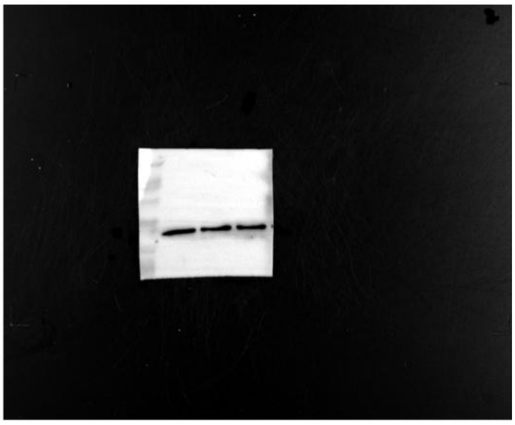

Biological replicate 2

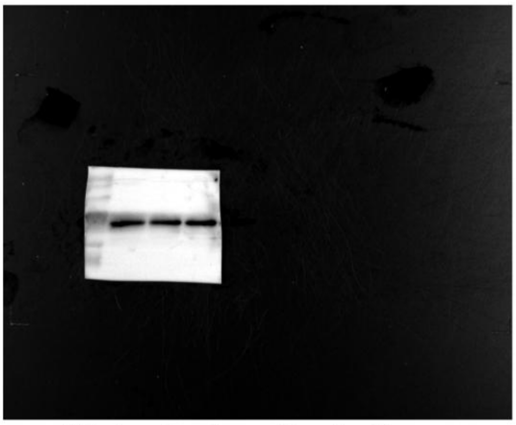

Biological replicate 3

Figure 2D occludin

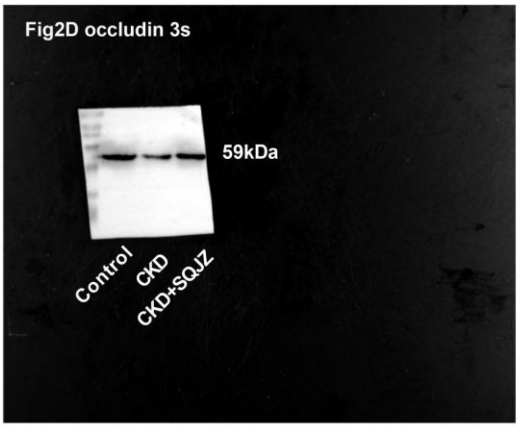

Representative blot

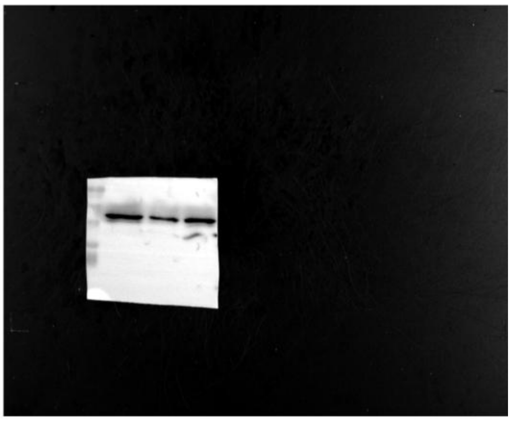

Biological replicate 2

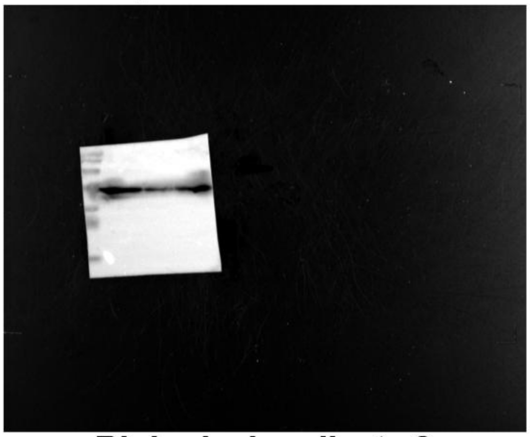

Biological replicate 3

Figure 5A CHAC1

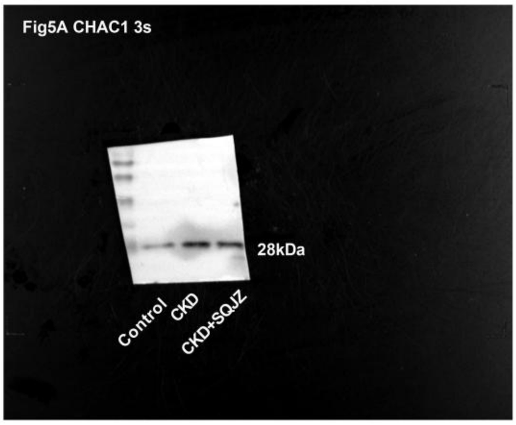

Representative blot

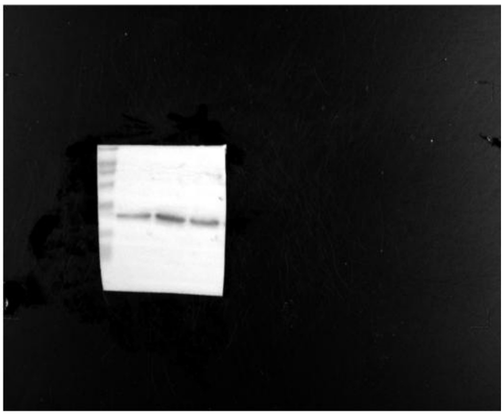

Biological replicate 2

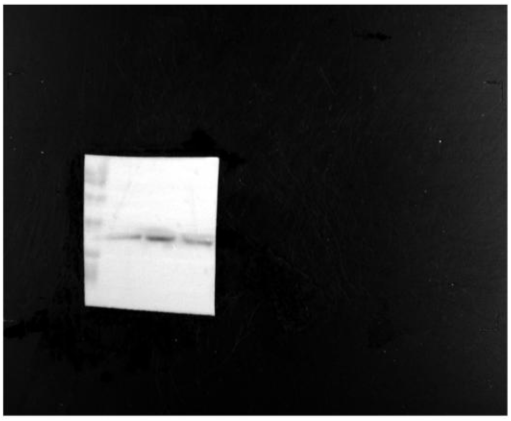

Biological replicate 3

Figure 5A GAPDH

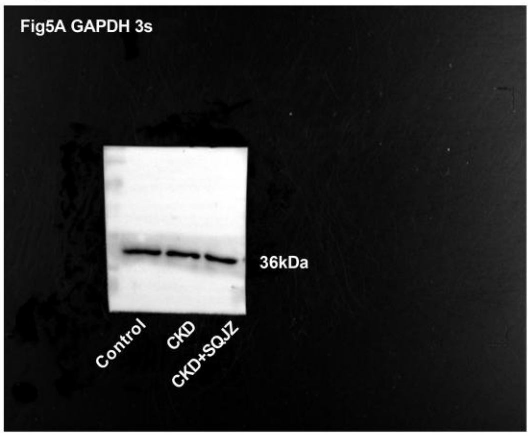

Representative blot

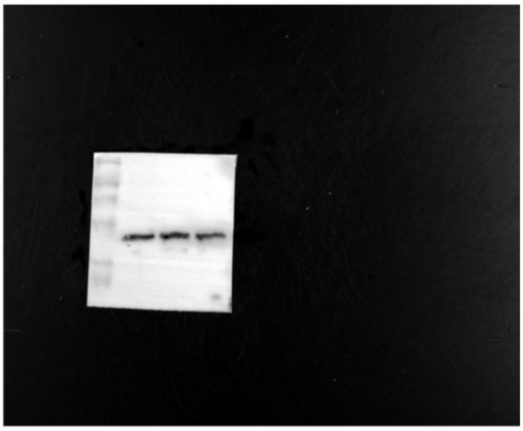

Biological replicate 2

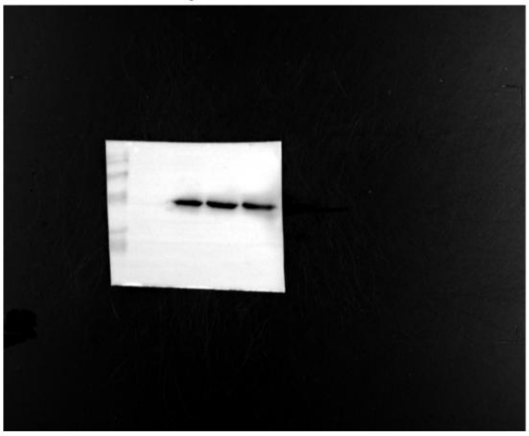

Biological replicate 3

Figure 5A HIF-1α

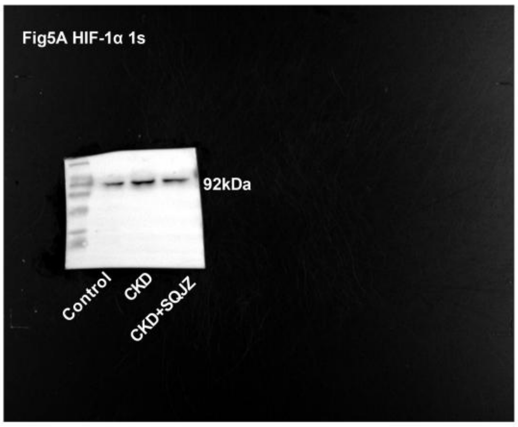

Representative blot

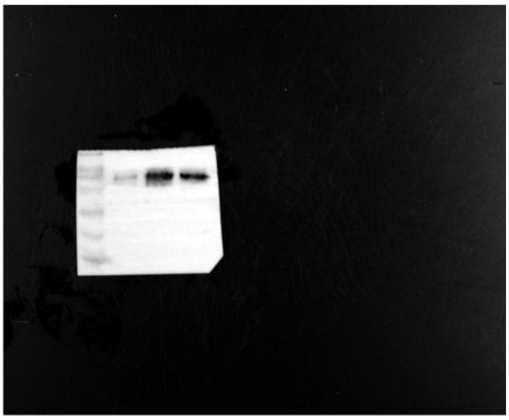

Biological replicate 2

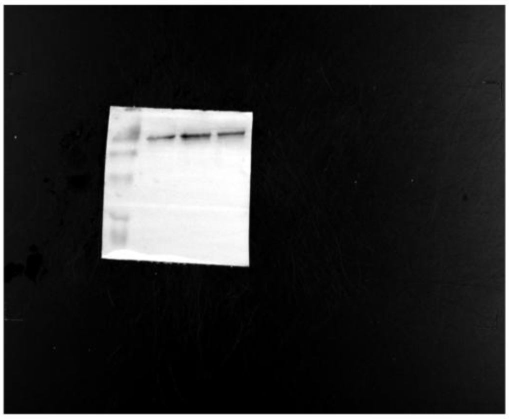

Biological replicate 3

Figure 5B GAPDH

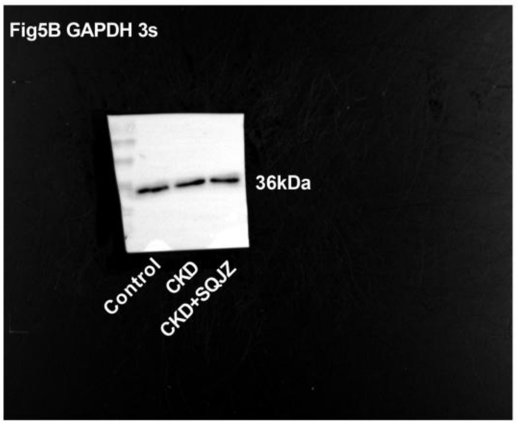

Representative blot

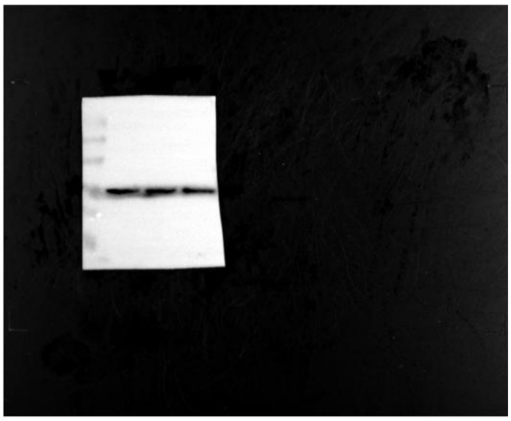

Biological replicate 2

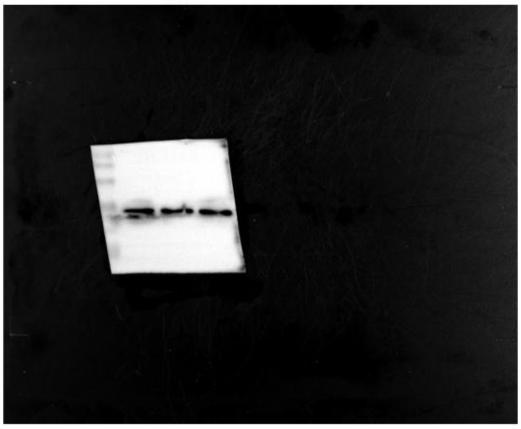

Biological replicate 3

Figure 5B GLUT1

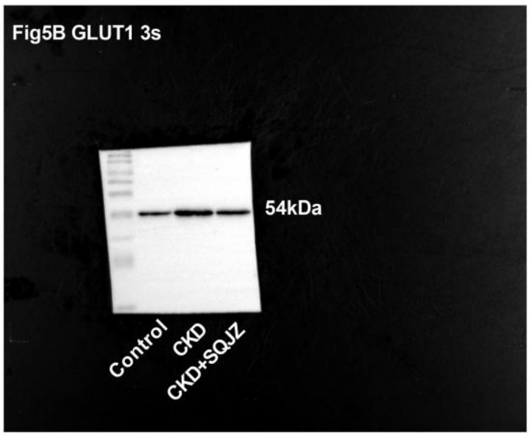

Representative blot

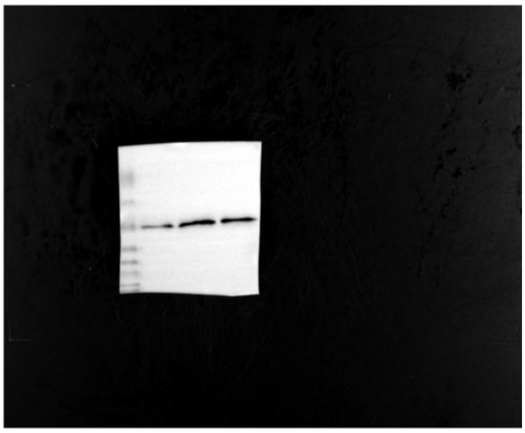

Biological replicate 2

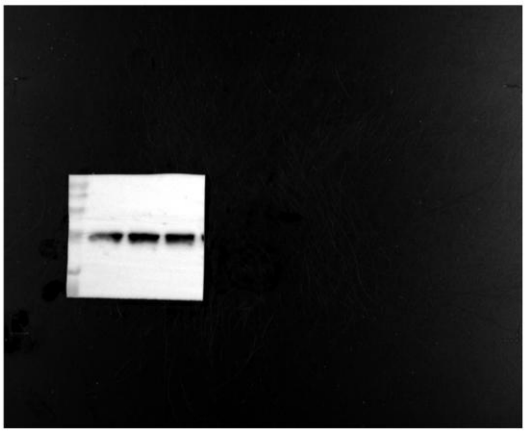

Biological replicate 3

Figure 5B VEGF

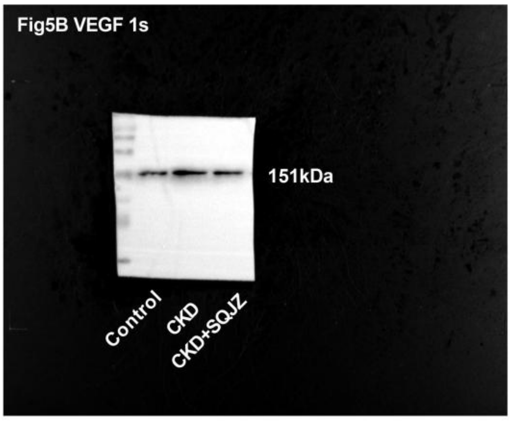

Representative blot

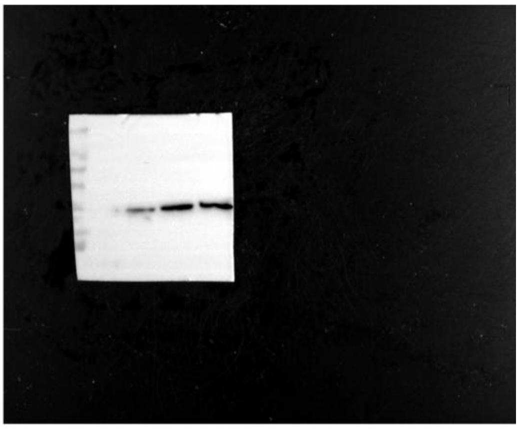

Biological replicate 2

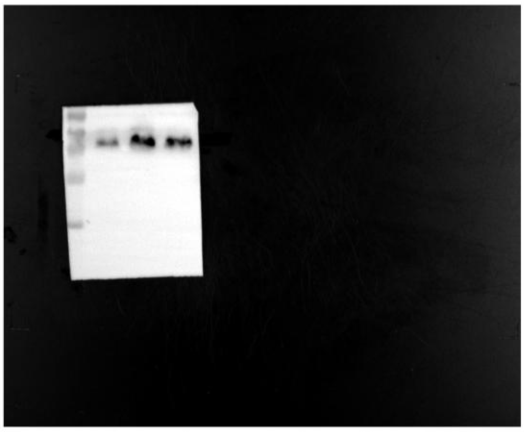

Biological replicate 3

Figure 6E CHAC1

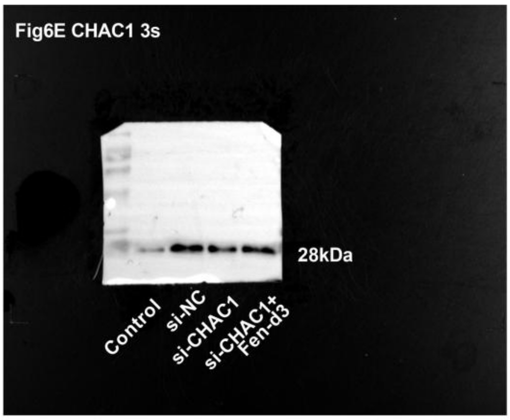

Representative blot

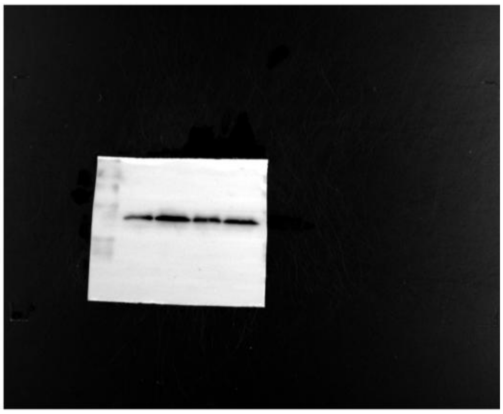

Biological replicate 2

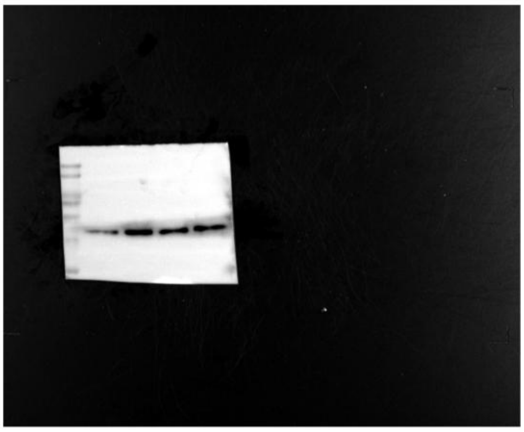

Biological replicate 3

Figure 6E GAPDH

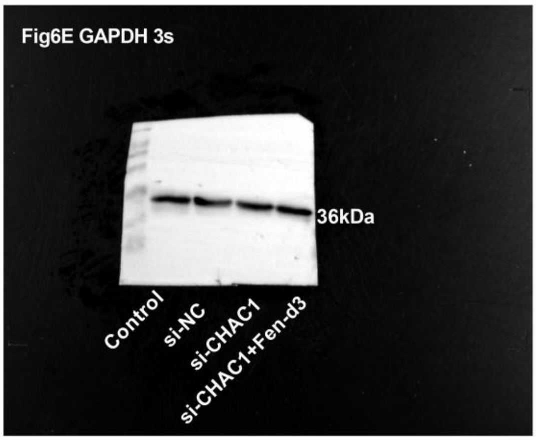

Representative blot

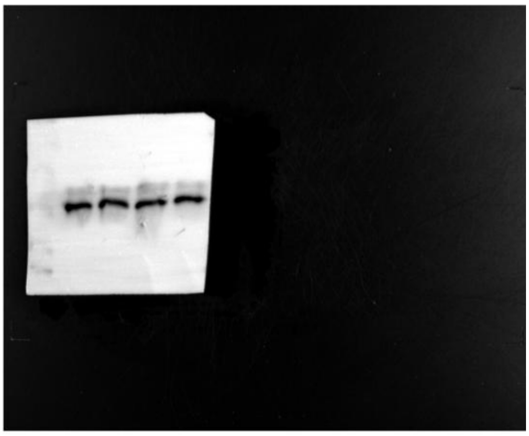

Biological replicate 2

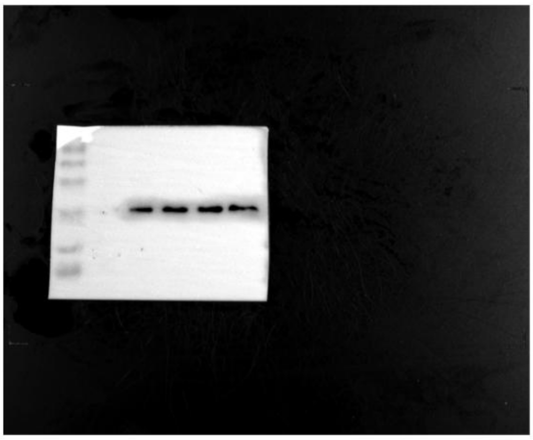

Biological replicate 3

Figure 6E HIF-1α

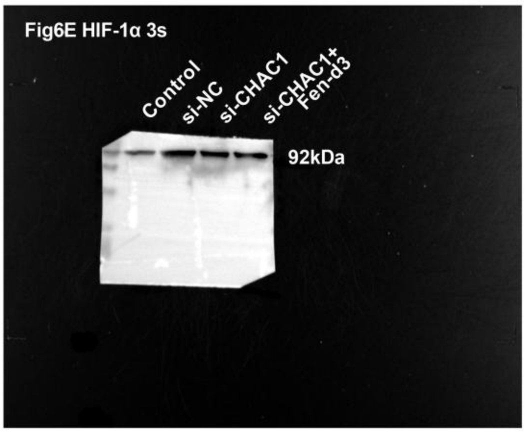

Representative blot

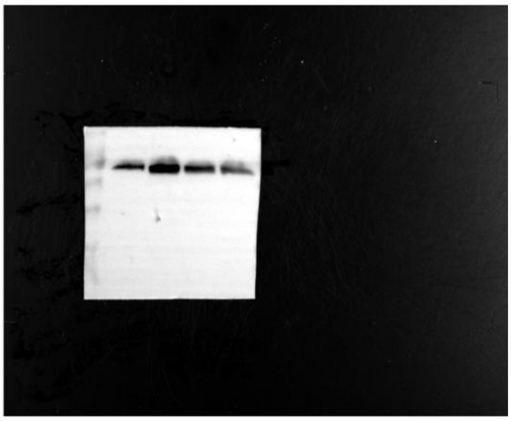

Biological replicate 2

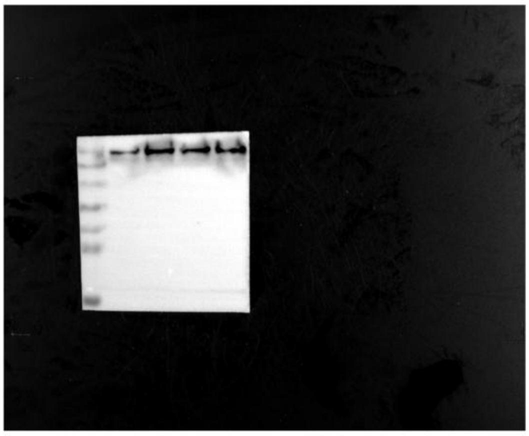

Biological replicate 3
